# Reduction of Bacterial Enteric Pathogens and Hygiene Indicator Bacteria on Tomato Skin Surfaces by a Polymeric Nanoparticle-Loaded Plant-Derived Antimicrobial

**DOI:** 10.3390/microorganisms10020448

**Published:** 2022-02-15

**Authors:** Keila L. Perez-Lewis, Yagmur Yegin, Jun K. Oh, Alejandro Castillo, Luis Cisneros-Zevallos, Chris R. Kerth, Mustafa Akbulut, Thomas M. Taylor

**Affiliations:** 1 Department of Food Science and Technology, Texas A&M AgriLife Research, College Station, TX 77843, USA; kperezlewis@gmail.com (K.L.P.-L.); yyegin@mit.edu (Y.Y.); alejandro.castillo@ag.tamu.edu (A.C.); 2 Artie McFerrin Department of Chemical Engineering, Texas A&M University, College Station, TX 77843, USA; junkyunoh@dankook.ac.kr (J.K.O.); makbulut@tamu.edu (M.A.); 3 Department of Horticultural Sciences, Texas A&M AgriLife Research, College Station, TX 77843, USA; lcisnero@tamu.edu; 4 Department of Animal Science, Texas A&M AgriLife Research, College Station, TX 77843, USA; c-kerth@tamu.edu

**Keywords:** tomato, sanitization, geraniol, encapsulation, *Salmonella typhimurium*, *E. coli* O157:H7, plant-derived antimicrobial, fruit decontamination, post-harvest

## Abstract

This study determined *Escherichia coli* O157:H7 and *Salmonella enterica* serovar Typhimurium survival on tomato skins as a function of sanitization treatment, under three differing contamination and sanitization scenarios. Sanitizing treatments consisted of the plant-derived antimicrobial (PDA) geraniol (0.5 wt.%) emulsified in the polymeric surfactant Pluronic F-127 (GNP), 0.5 wt.% unencapsulated geraniol (UG), 200 mg/L hypochlorous acid at pH 7.0 (HOCl), and a sterile distilled water wash (CON). Experimental contamination and sanitization scenarios tested were: (1) pathogen inoculation preceded by treatment; (2) the pathogen was inoculated onto samples twice with a sanitizing treatment applied in between inoculations; and (3) pathogen inoculation followed by sanitizing treatment. Reductions in counts of surviving pathogens were dependent on the sanitizing treatment, the storage period, or the interaction of these independent/main effects. GNP treatment yielded the greatest reductions in pathogen counts on tomato skins; pathogen survivor counts following GNP treatment were consistently statistically lower than those achieved by HOCl or UG treatments (*p* < 0.05). GNP treatment provided greatest pathogen reduction under differing conditions of pre- and/or post-harvest cross-contamination, and reduced hygiene-indicating microbes the most of all treatments on non-inoculated samples. Encapsulated geraniol can reduce the risk of pathogen transmission on tomato fruit, reducing food safety hazard risks for tomato consumers.

## 1. Introduction

The U.S. Department of Agriculture reports that outbreaks of human foodborne microbial disease resulting from consuming pathogen-contaminated tomatoes have declined in frequency since 2001, but have nonetheless been a consistent annual occurrence in the U.S. [1]. Gurtler et al. [2] reviewed multiple disease outbreaks involving transmission of *Salmonella* serovars via tomatoes resulting in hundreds of disease cases. From August to October 2019, an outbreak of salmonellosis in Sweden occurred from imported contaminated tomatoes yielding 82 confirmed cases of disease [3]. These and other outbreaks continue to present challenges to food safety protection efforts for consumers of fresh and fresh-cut tomatoes. While *Salmonella* has been more frequently linked to disease outbreaks resulting from tomato contamination, distribution and ultimate consumption, researchers have demonstrated the capacity of tomatoes to support *Escherichia coli* O157:H7 contamination in production and subsequent transmission on harvested fruit [4]. The development and application of tomato sanitization interventions is thus warranted to reduce the likelihood of pathogen transmission and consequent disease that might otherwise occur.

Much research has been conducted already describing the decontamination of pathogens and other microbes from tomato surfaces using various chemical and biological antimicrobial interventions. Gurtler et al. [2] expertly reviewed many research reports, detailing differences in *Salmonella* reductions observed on tomatoes as a function of sanitizing chemical concentration and application conditions. Recent research has explored the use of plant-derived antimicrobials (PDAs) delivered to tomatoes to achieve pathogen reductions using a variety of means of application, including novel technologies such as nano-emulsion and edible film coatings. Zhang et al. [5] recently reported the use of thymol (0.4 mg/mL) produced low level reductions on cut tomatoes of both *E. coli* O157:H7 and *Staphylococcus aureus* cells. Ruengvisesh et al. [6] applied eugenol-loaded micelles constructed of sodium dodecyl sulfate (SDS) to *S. saintpaul* and *E. coli* O157:H7-inoculated Roma tomatoes that were then held refrigerated for up to 10 days. Pathogens were reduced by ~5.0 log_10_-cycles by application of eugenol, either naked or loaded into micelles, outperforming 200 ppm HOCl. In addition, numbers of aerobic bacteria and *Enterobacteriaceae* were reduced to the limit of detection (0.5 log_10_ CFU/cm^2^). He et al. [7] produced similar reductions in numbers of cherry tomato-contaminating *E. coli* O157:H7 following application of thymol-loaded micelles constructed of the cationic surfactant cetylpyridinium chloride (CPC).

Kirkland et al. [8] reported on best practices for handling of tomatoes in food service kitchens for food safety protection; the researchers reported that the use of chlorine or other sanitizer occurred in a small number of observed instances amongst studied restaurants. The use of sanitization treatments, including encapsulated PDAs, in concert with other best practices for consumer and food service kitchen food safety protection, is a necessary practice to ensure maximal pathogen decontamination prior to consumption. Thus, the primary objective of this research was to compare the utility of PDA-loaded nanoparticles constructed of the amphiphilic co-polymer Pluronic F-127 to decontaminate Roma tomato skins from the pathogens *Salmonella typhimurium* and *E. coli* O157:H7 versus other sanitization treatments as a function of the sequence of contamination and sanitization treatments. This was done to determine whether antimicrobial nanoparticles can provide extended pathogen inhibition in situations where good agricultural practices (GAPs) during post-harvest handling of tomatoes were not strictly followed.

## 2. Materials and Methods

### 2.1. Preparation of Microorganisms for Tomato Sample Inoculation

Isolates of *E. coli* O157:H7 (ATCC 700728) and *Salmonella enterica* serovar Typhimurium (ATCC 700720) were revived from cryo-storage (−80 °C) by duplicate sequential passes in tryptic soy broth (TSB; Becton, Dickinson and Co., Sparks, MD, USA) with incubation at 37 °C for 24 h after each pass. Both isolates were verified as naturally resistant to rifampicin (Rif^+^) by plating on tryptic soy agar supplemented with 100.0 µg/mL rifampicin (TSAR; Becton, Dickinson and Co.) and then incubating plates for 24 h at 37 °C before checking for colony development. After reviving and verifying Rif^+^ for each organism, a cocktail of both organisms for inoculation onto tomato skin samples was prepared using centrifugation conditions reported in Perez-Lewis et al. [9], producing an inoculum prepared to a target of 8.0 log_10_ CFU/mL for use on tomato skin samples.

### 2.2. Preparation of Tomato Skin Samples for Inoculation by Pathogen Cocktail

Non-waxed Texas-grown Roma tomatoes at differing degrees of ripeness were purchased from a College Station, TX, fruit and vegetable wholesaler and transported to the Food Microbiology Laboratory (FML) (Department of Animal Science, Texas A&M University, College Station, TX, USA). After arriving at the FML, tomatoes were washed in sterile distilled water and placed over sterilized steel racks to dry for 1 h at ambient condition. Following drying, a flame-sterilized stainless-steel borer and forceps were used to excise 10 cm^2^ tomato skin sample discs (~2 mm depth for each disc). For each sample, three discs were placed together in a sterile plastic dish for subsequent inoculation and sanitization treatment (30 cm^2^ total sample surface area).

### 2.3. Tomato Sample Inoculation by Cocktailed Pathogens

Tomato samples were inoculated by spot-inoculation of cocktailed *E. coli* O157:H7 and *S.* *typhimurium* cells using the same method previously reported [9,10]. Briefly, 0.1 mL total inoculum fluid was applied by evenly distributing ten 10.0 μL spots over the three combined tomato discs within a sample. After spotting inoculum onto tomato skin samples, samples were placed in a biological safety cabinet for 1 h to facilitate inoculum fluid evaporation and microbial inoculation attachment to tomato sample surfaces.

### 2.4. Tomato Inoculation and Sanitization Treatment Scenarios

For tomato samples inoculated with cocktailed pathogens, three contamination and sanitization scenarios were tested to determine whether the sequence of pathogen inoculation (contamination) and application of sanitization treatment impacted observed pathogen reduction/survival. Contamination/sanitization scenarios were conducted in identical fashion as we recently described for similar experiments on muskmelons [9]. Scenarios were:

1. Scenario 1: The mixed *E. coli* O157:H7 and *S. typhimurium* organisms were inoculated prior to sanitization treatment, simulating contamination occurring immediately prior to or during tomato harvest;
2. Scenario 2: Pathogens were inoculated twice onto tomato samples, once before sanitization treatment and then again after 3 days of refrigerated storage, simulating conditions of pathogen contamination both during harvest and again during post-harvest packing, and;
3. Scenario 3: Pathogens were inoculated/contaminated onto already-treated tomato samples, wherein tomato samples were treated by one of four sanitization treatments (Section 2.5), covered, and then placed under refrigeration (5 °C) for 3 days. On day 3, samples were removed from refrigeration, inoculated with cocktailed pathogens, and then returned to refrigerated storage or immediately prepared for enumeration of surviving cells.

### 2.5. Preparation and Application of Sanitization Treatments to Tomato Sample Surfaces

Antimicrobial essential oil component-containing particles composed of the triblock co-polymer Pluronic F-127 loaded with the PDA geraniol (GNPs) (CAS #106-24-1; TCI America, Portland, OR, USA; >98%) were prepared as previously described [11,12]. Geraniol-loaded particles were formulated to contain 0.5 wt.%. Unencapsulated geraniol (UG) was prepared in sterile distilled water to 0.5 wt.%. A chlorine treatment consisting of 200 mg/L hypochlorous acid (HOCl; pH 7.0 ± 0.1) was prepared to provide sanitizer treatment used commonly in fresh fruit and vegetable washing, and then finally a control (CON) treatment consisting of washing with sterile distilled water was included.

Treatments were applied as described previously [10], where samples were exposed to sanitizer treatment by immersing samples in 20 mL of sanitizer fluid for 2 min; after exposure, sanitizer fluid was aseptically removed and samples placed on sanitary paper towels for 15 min to allow remaining sanitizer fluid to drip off. Afterwards, samples were transferred to sterile stomacher pouches for microbiological analysis, or placed into sterile plastic dishes, covered with an oxygen-permeable film (LDPE), and placed at 5 °C for 3, 5, 7, or 10 days. For all samples processed under Scenario 2, the first pathogen inoculation was completed using an inoculum prepared to a target of 8.0 log_10_ CFU/mL (identical to that prepared for use with samples assigned to Scenario 1 and 3 experiments). The second pathogen contamination/inoculation event occurred after 3 days of refrigerated storage, applying an inoculum prepared to 7.0 log_10_ CFU/mL according to previous risk assessment of post-harvest fruit and vegetable pathogen contamination [13]. Finally, Scenario 3-assigned samples were inoculated after 3 days of refrigerated (5 °C) storage by spot-inoculating with an inoculum fluid prepared to a target of 8.0 log_10_ CFU/m (Section 2.1).

### 2.6. Testing of Sanitization Treatments Efficacy against Hygiene Bacterial Organisms on Non-Inoculated Tomato Skin Samples

In addition to pathogen cocktail-inoculated tomato samples, a set of identically prepared (Section 2.2) but not inoculated tomato skin samples were treated with sanitization treatments in identical fashion as described for Scenario 1 above (Section 2.4). Following sanitization treatment, samples were either processed for microbiological analysis of aerobically growing bacteria, total lactic acid bacteria (LAB), or total coliforms on appropriate media to ascertain the impact of sanitization treatment on resulting numbers of surviving hygiene indicating bacteria as a function of days of storage at 5 °C. In addition to microbiological analyses, photographs were collected of treated tomato samples from samples subjected to differing sanitization treatments at each storage period (0, 3, 5, 7, or 10 days) to determine impacts on tomato sample appearance by treatment and storage [11].

### 2.7. Microbiological Analysis of Inoculated or Uninoculated Tomato Samples

Microbiological analyses were carried out as reported previously [6]. Briefly, *E. coli* O157:H7 and *S. typhimurium* were enumerated from tomato samples by placing a sample in a stomacher pouch containing 99 mL sterile 0.1% (*w*/*v*) peptone diluent (Becton, Dickinson and Co.), followed by pulverizing at 260 rpm for 1 min. Decimal dilutions were prepared in 0.1% peptone diluent and spread on surfaces of lactose sulfite phenol red rifampicin (LSPR) agar containing 100 μg/mL rifampicin [10]. Rifampicin was prepared in absolute methyl alcohol and then added to sterilized, tempered agar base, swirled to mix, poured into 100 × 15 mm sterile Petri dishes, and allowed to cool and gelatinize prior to inoculation. The limit of detection (LOD) for enumeration of surviving pathogen cells was 0.5 log_10_ CFU/cm^2^. Pathogen cells were enumerated after 24 h incubation at 36 ± 1 °C incubation.

For uninoculated tomato samples, aerobic bacteria were enumerated on 3M™ Petrifilm™ Aerobic Count Plate films following preparation of decimal dilutions in 0.1% peptone diluent and inoculation. Inoculated films were incubated at 36 ± 1 °C for 48 h prior to colony counting per manufacturer guidance. The LAB were enumerated by preparing decimal dilutions of tomato samples in de Man, Rogosa, and Sharpe (MRS) broth (Becton, Dickinson and Co.), then inoculating onto Aerobic Count Plate petrifilms, and finally incubating films for 48 h at 36 ± 1 °C prior to colony counting. Coliforms were enumerated from a 3M™ Petrifilm™ *E. coli*/Coliform Count Plate following incubation at 36 ± 1 °C for 48 h.

### 2.8. Experimental Design and Statistical Analysis

Tomato samples subjected to sanitizing treatment (pathogen-inoculated or uninoculated) were designed as a factorial arrangement with a complete randomized design. Main effects for both inoculated and non-inoculated sets of samples were the sanitizing treatment and storage period. All experiments were replicated thrice identically (*n =* 3). Microbiological counts of *E. coli* O157:H7 and *S. typhimurium* on inoculated samples log_10_-transformed prior to analysis and *E. coli* O157:H7-specific data were not statistically compared with *S. typhimurium* data. Two-way analysis of variance (AOV) testing the main effects and their interaction was performed using GraphPad Prism v.9.3 for macOS (GraphPad Software, San Diego, CA, USA). Post-hoc testing was completed using Tukey’s Honestly Significant Differences (HSD) test, with means considered significantly different at *p* < 0.05. Similarly, log_10_-transformed microbiological data for aerobic bacteria (i.e., aerobic plate count), LAB, and total coliforms were subjected to 2-way AOV and Tukey’s HSD testing to identify impacts of main effects of treatment and storage period, or their interaction, on resulting counts of these organisms on tomato samples. Lastly, the interaction of sanitizing treatment (GNP, UG, HOCl, CON) by the experimental scenario (1, 2, 3) was analyzed to determine if any of the four treatments consistently produced lower counts of surviving pathogens across the three contamination and sanitization processes versus other treatments, similar to our recent report on similarly treated melons [9].

## 3. Results

### 3.1. Reduction in Bacterial Pathogen Survivors on Tomato Samples from Differing Contamination and Sanitization Scenarios

#### 3.1.1. Pathogen Contamination on Tomatoes Precedes Sanitization Treatment (Scenario 1)

**Figure 1:**
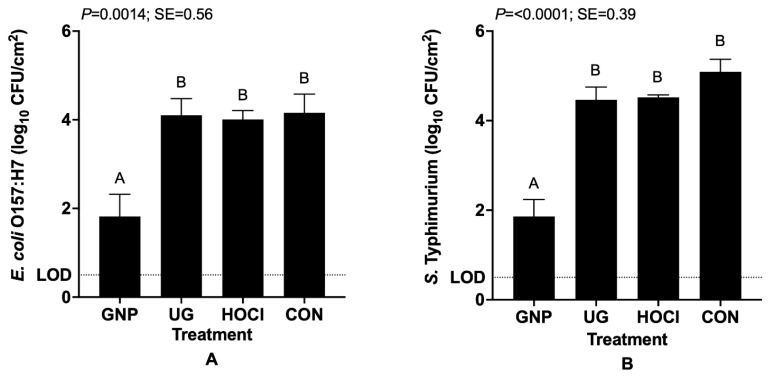
Least squares means of surviving *E. coli* O157:H7 (**A**) and *S. typhimurium* (**B**) cells on inoculated and treated tomato samples. Treatments are GNP (0.5 wt.% geraniol in polymeric nanoparticles), UG (0.5 wt.% unencapsulated geraniol in sterile distilled water), HOCl (200 mg/L pH 7.0 hypochlorous acid), CON (sterile distilled water). Bars represent means of triplicate identical replicates (*n* = 3); error bars depict the standard error of means. SE: pooled standard error. Bars not sharing a capitalized letter (A, B) differ at *p* = 0.05 by one-way analysis of variance and Tukey’s Honestly Significant Differences test.

Figure 1 depicts the survival of *E. coli* O157:H7 (Panel A) and *S. typhimurium* (Panel B) on tomato samples after inoculation and then subsequent sanitization treatment with one of the food-sanitizing treatments. For tomato samples subjected to contamination prior to sanitization, two trends are evident from survivor count data. The first occurring for both pathogens is that despite inoculating with a cocktail prepared to a mean of 7.6 ± 0.1 and 7.6 ± 0.14 log_10_ CFU/mL for *E. coli* O157:H7 and *S. typhimurium*, respectively, a relatively efficient inoculation was achieved. The 0.1 mL inoculum was distributed over a total of 30 cm^2^, delivering approximately 6.8 ± 0.2 log_10_ CFU/cm^2^; dividing by 30 cm^2^ yielded a resulting predicted inoculum of ~5.2 log_10_ CFU/cm^2^. Indeed, both pathogens on day 0 samples were inoculated to 5.7 ± 0.4 log_10_ CFU/cm^2^ on CON-treated sample surfaces (data not shown). The second trend observable for each organism was that, although the interaction of sanitization treatment by storage period did not significantly influence resulting plate counts of surviving cells, the GNP treatment produced a significantly lower number of surviving pathogen cells (~1.8 log_10_ CFU/cm^2^ for each organism) versus all other treatments (*p <* 0.05) (Figure 1).

#### 3.1.2. Pathogen Survival When Contamination of Tomatoes Both Precedes and Follows Sanitization Treatment (Scenario 2)

**Figure 2:**
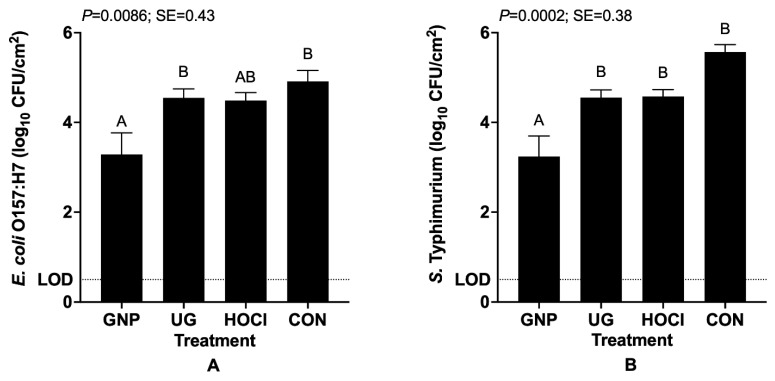
Least squares means of surviving numbers of *E. coli* O157:H7 (**A**) and *Salmonella typhimurium* (**B**) on tomato skin samples when inoculated with pathogens before and after sanitization treatment. Treatments are GNP (0.5 wt.% geraniol in polymeric nanoparticles), UG (0.5 wt.% unencapsulated geraniol in sterile distilled water), HOCl (200 mg/L pH 7.0 hypochlorous acid), CON (sterile distilled water). Bars represent means of triplicate identical replicates (*n* = 3); error bars depict the standard error of means. SE: pooled standard error. Bars not sharing a capitalized letter (A, B) differ at *p* = 0.05 by one-way analysis of variance and Tukey’s Honestly Significant Differences test.

Figure 2 depicts the survival of pathogenic organisms when inoculation of pathogens occurred both before and after sanitization treatment, thus simulating the potential for pathogen contamination to occur both during harvest and after initial washing in post-harvest packing, due either to insanitary surfaces or worker hygiene failure. As with the data above (Section 3.1.1) demonstrating the impact of treatment on pathogen survival following sanitization, data analysis did not identify a significant interaction of treatment × storage period effects. GNP treatment again was most effective at lowering the numbers of surviving pathogen cells versus the CON treatment, producing the only statistically differing survivor counts for treatments tested. Inoculation efficacy on samples was similar as for Scenario 1 data, and surprisingly the second inoculation with organisms (6.8 ± 0.25 log_10_ CFU/mL) did not produce an appreciable increase in numbers of cells on tomato surfaces, though increases in counts of both organisms are not evident in Figure 2 given the presentation of only the survivor counts by treatment, rather than by storage period.

#### 3.1.3. Pathogen Survival on Tomatoes When Contamination Follows Sanitization Treatment (Scenario 3)

**Figure 3:**
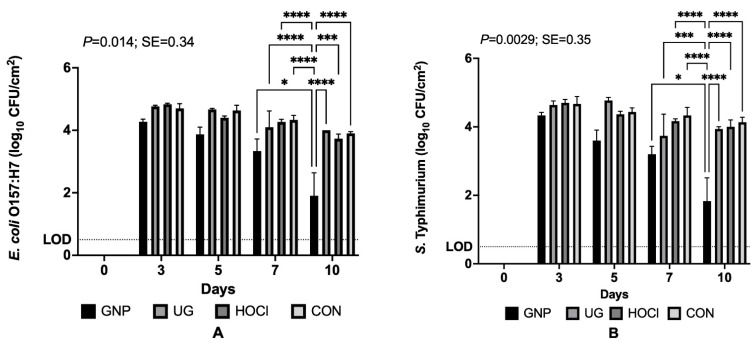
Least squares means of *E. coli* O157:H7 (**A**) and *Salmonella typhimurium* (**B**) on tomato skins from the interaction of sanitization treatment × number of days stored at 5 °C. Bars represent means from triplicate identical replicates (*n* = 3); error bars depict the standard error of means. SE = pooled standard error. Tomato samples were treated by sanitizer treatment, placed at 5 °C for 3 days and then inoculated, and then returned to 5 °C storage. GNP: 0.5 wt.% loaded polymeric nanoparticles; UG: unencapsulated 0.5 wt.% geraniol in sterile water; HOCl: 200 mg/L pH 7.0 hypochlorous acid; CON: sterile distilled water. Bars connected by difference indicators differ at: * (*p* < 0.05), *** (*p* < 0.001), or **** (*p* < 0.0001).

In contrast to Scenarios 1 and 2, for tomato samples inoculated 3 days after treatment, the interaction of treatment with storage period did significantly influence resulting microbiological survivor data/results. Figure 3 below demonstrates again that GNP treatment yielded the best results in terms of pathogen reductions, producing survivor counts that mirrored those of results obtained for Scenario 1 (Section 3.1.1). As shown in Figure 3, immediately upon inoculation at day 3 of storage following sanitizer treatment on day 0, all inoculated tomato samples bore between 4.0 and 5.0 log_10_ CFU/cm^2^, and consistently across treatments the numbers of surviving cells declined in refrigerated storage at 5 °C through the experiment’s end. Nonetheless, between days 7 and 10, significant declines in survivor counts, while occurring for all treatments, were pronounced for GNP-treated tomato samples, declining by more than a log_10_-cycle, resulting from the continued release of geraniol from slowly degrading encapsulates and the impacts of cold storage on slowing bacterial growth and respiration [12].

### 3.2. Antimicrobial Impacts of Sanitization Treatment by Experimental Scenario for Pathogen-Inoculated Tomatoes

Table 1 and Table 2 below depict the results of data analysis for the interactions of sanitization treatment (GNP, UG, HOCl, CON) by the tomato sample contamination and sanitization scenario (1, 2, or 3). For both *E. coli* O157:H7 and *Salmonella typhimurium* survivor counts on sample surfaces, the GNP treatment reduced numbers of surviving cells to a lower count than did other sanitization treatments, apart from certain comparisons for *S. typhimurium* counts (Table 2). For *E. coli* O157:H7, counts on GNP-treated samples were lower regardless of treatment scenario, suggesting enhanced performance of the encapsulated geraniol for pathogen growth inhibition. For *S. typhimurium*, the GNP treatment in Scenario 3 was numerically lower than other treatments, but not statistically so when compared to UG- and HOCl-treated samples. Nonetheless, data analyses indicate GNP treatment performed the best for purposes of lowering the numbers of surviving pathogens on tomatoes regardless of the sequence of tomato contamination and sanitization events during harvest and/or post-harvest handling/packing.

### 3.3. Antimicrobial Impacts of Sanitizer Treatments on Tomato Hygiene Indicator Bacteria Groups for Tomato Samples Not Inoculated with Pathogens

#### 3.3.1. Pathogen Contamination on Tomatoes Precedes Sanitization Treatment

For tomato samples not inoculated with *E. coli* O157:H7 and *S. typhimurium*, the application of sanitization treatments under conditions similar to Scenario 1 resulted in reductions in the numbers of microbes that trended similarly to those reported for pathogen reductions above (Section 3.1.1). Table 3 presents the survival of aerobic bacteria, LAB, and coliforms as a function of sanitization treatment; like those results obtained for pathogen-inoculated tomato skin samples, there was not a significant interaction of treatment by storage period for data (*p* ≥ 0.05). Tomatoes, despite being washed prior to inoculation, still bore elevated numbers of both aerobically growing and lactic acid-fermenting bacteria as indicated on CON samples, in addition to total coliforms, indicating a trending towards microbiological quality loss. Nevertheless, as was observed for pathogen-inoculated experiments, the GNP treatment produced the greatest reductions in numbers of surviving pathogens when comparing the GNP treatment to the CON treatment for all microbe groupings. Statistical differences between means of sanitization treatments (specifically GNP versus UG and/or HOCl) were less frequent than was observed during pathogen-inoculation experiments, with only LAB demonstrating a statistically lower count of surviving LAB following GNP treatment versus UG or HOCl treatment (Table 3). GNP was the only treatment to produce at least a 2.0 log_10_-cycle decline in surviving bacteria counts for each grouping when compared to the CON treatment; differences ranged from 2.2 to 2.4 log_10_-cycles. UG and HOCl treatments produced only modest reductions in surviving populations, ranging from 0.9 log_10_ CFU/cm^2^ when comparing the CON and HOCl treatment means for APC and LAB, to 1.8 log_10_ CFU/cm^2^ for the difference in coliforms treated by CON versus UG (Table 3).

#### 3.3.2. Impact of Sanitization Treatment on Tomato Skin Appearance during Storage

**Figure 4:**
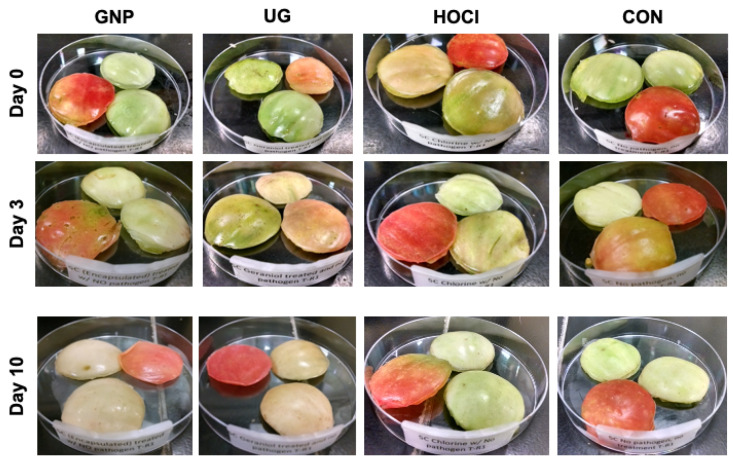
Changes in tomato skin appearance following sanitization treatment. GNP (0.5 wt.% geraniol in polymeric nanoparticles), UG (0.5 wt.% unencapsulated geraniol in sterile distilled water), HOCl (200 mg/L hypochlorous acid), CON (sterile distilled water).

Figure 4 depicts post-treatment appearance changes occurring on tomato skin samples over 10 days of refrigerated (5 °C) storage. Images for tomato samples at 5 and 7 days of storage are not presented due to little evident change in sample appearance between days 3 and 10 (data not shown). None of the sanitization treatments provided apparent protection against color loss in tomato samples. GNP- and UG-treated tomatoes appeared dehydrated near the edges of sample discs by day 10 of refrigerated storage, like the appearance of HOCl- or CON-treated samples at 10 days’ refrigerated storage.

## 4. Discussion

In the current manuscript, like our group’s previous publications on aligned research investigating encapsulated geraniol as a PDA effective for pathogen decontamination on spinach and melon rind, encapsulated geraniol (0.5 wt.%) was applied to tomato skin samples and compared for its antimicrobial activity during refrigerated storage lasting 10 days [9,10,11]. As was the case in those studies, the GNP treatment consistently yielded lower numbers of surviving *E. coli* O157:H7 and *Salmonella typhimurium* cells on treated tomato samples as compared to unencapsulated PDA or 200 mg/L hypochlorous acid treatment. Across the three contamination and sanitization scenarios, GNPs demonstrated enhanced capacity to inhibit the growth of pathogens, in particular Scenario 3 (Figure 3); GNPs provided greater long-term suppression of pathogen growth than any other treatment. Like our group’s results when testing these sanitizing treatments on spinach leaves, interactions of main effects were not detected for Scenario 1-type data here (Section 3.1.1) but only the main effect of treatment significantly impacted resulting microbiological data (Figure 1). Additionally, while the numbers of both pathogens on tomato samples in the current study were similar to the numbers of organisms on melon samples subjected to the same CON treatment as described here (Section 2.5), the reductions achieved by sanitization treatments for tomatoes were numerically greater than those for cantaloupes/melons, potentially due to differences in attachment capability of organisms when encountering the smooth, more hydrophobic surface of tomato skin versus melon rind netting [9,14]. Similar outcomes were observed when comparing the current study data from Scenario 2 (contamination, sanitization, then re-contamination) for melons and tomatoes, wherein the recontamination event at day 3 of refrigerated storage lessened the observed reductions in pathogen survival versus other scenarios not using two sequential pathogen inoculation/contamination steps. Interestingly, reductions achieved in the current study on tomatoes were also greater than those obtained for melons for Scenario 2, again likely due to differences in attachment capacity of organisms to produce commodities with highly differing surface physico-chemistries.

Data obtained within Scenario 1 experiments indicate that GNPs performed optimally among the tested sanitization treatments, producing greater reductions in numbers of surviving pathogens than chlorine or non-encapsulated PDA treatments reported by some researchers applying sanitizers at concentrations approximately equal to those used in the current study on fresh and fresh-cut tomatoes [5,15]. Gurtler et al. [2] reviewed the literature on tomato decontamination, describing the use of 200 ppm (mg/L) HOCl as inconsistently producing near complete removal of countable salmonellae from tomato surfaces, but not consistently able to decontaminate *Salmonella* attaching to stem scar or lenticel sites on tomato fruit. In the current study (Figure 1), HOCl treatment produced non-differing numbers of surviving *E. coli* O157:H7 and *S. typhimurium* from the CON, indicating its primary utility remains maintaining against wash water decontamination rather than direct disinfection of cross-contaminated harvested fruit. GNP treatment in the current study also out-performed other reported tomato sanitizing interventions. Application of 2.0 or 3.0 μg ozone/g tomato fruit to *E. coli* O157:H7 and *Listeria monocytogenes*-contaminated tomatoes resulted in a maximum 1.5 log_10_-cycle reduction for both pathogens following 2–3 h application [16]. Electrolyzed water was assessed to produce reductions of 1.98–2.0 log_10_-cycles in addition to those produced by water washing for pathogens on tomato surfaces, similar to the differences in reductions produced here for the GNP versus CON treatments [17]. Abuladze et al. [18] reported a reduction in *E. coli* O157:H7 numbers on tomato flesh of 95–99% (approximately 1.8–2.0 log_10_-cycles) versus controls when treated with infectious bacteriophages. On the other hand, 2% lactic or malic acid application reduced *E. coli* O157:H7 on cherry tomato surfaces slightly more effectively, achieving 2.3–2.5 log_10_ CFU/g. Assays carried out testing *Salmonella typhimurium* decontamination on cherry tomatoes yielded even more impressive results from these organic acids: 4.3–4.5 log_10_ CFU/g reductions in the pathogen counts [19].

Previous research on the decontamination of *Salmonella* and/or *E. coli*-inoculated tomatoes using plant-derived essential oil components has reported pathogen reductions similar to those reported herein where pathogens were inoculated prior to sanitization treatment, similar to Scenario 1 in the current study. Gündüz et al. [20] tested the application of sumac and oregano oil preparations against *S. typhimurium* on inoculated tomatoes, varying the concentration and time of exposure to the essential oil component prior to determining pathogen survival. Application of 1% sumac oil for 5 min produced a 1.05 log_10_-cycle reduction in *Salmonella typhimurium* cells, roughly twice that observed for unencapsulated geraniol applied at 0.5 wt.% on tomatoes subjected to Scenario 1 testing (Figure 1B). Other researchers reported much larger apparent reductions of *Salmonella* serovars inoculated onto plum tomatoes following 1 min of washing in 0.75% eugenol, although pathogen reductions were reported in log_10_ CFU/mL of washing/dilution fluid rather than per cm^2^ of inoculated tomatoes [21]. Lu et al. [22] reported the combination of 0.2% thymol loaded into 4% SDS micelles produced a ~2.4 log_10_ CFU/g reduction in *Salmonella* inoculated onto grape tomatoes, similar to that obtained herein for GNP-treated Roma tomatoes (Figure 1B). Our research group recently reported 1.0% eugenol-loaded SDS micelles or 1.0% non-encapsulated eugenol reduced *S. typhimurium* and *E. coli* O157:H7 to near the limit of detection (0.5 log_10_ CFU/cm^2^) within hours of treatment, and protected tomatoes against statistically significant growth for 10 days of refrigerated post-treatment storage [6]. Nonetheless, SDS micelles did not outperform either free eugenol or 200 mg/L HOCl in those experiments with respect to observed pathogen reductions.

The testing of the experimental Scenario 2, incorporating two pathogen contamination events surrounding sanitization treatment, has not been extensively reported in the literature, as it presumes a gross failure in good agricultural practices (GAPs) for the preservation of microbiological safety, and best practices during food crop production and post-harvest packing/handling. Our previous publications on melons demonstrated the efficacy of GNP and UG treatments to produce statistically significant, albeit numerically small, reductions in numbers of both *E. coli* O157:H7 and *S. typhimurium* [9]. In the current study, reductions in pathogen counts were lower for *E. coli* O157:H7 versus *S. typhimurium*. *E. coli* O157:H7 survivor numbers were reduced only by 0.5–1.3 log_10_ CFU/cm^2^ in a treatment-dependent fashion, whereas for *S. typhimurium* reductions were larger: ~1.0–2.4 log_10_ CFU/cm^2^ (Figure 2). As discussed above, physico-chemical and topographical differences between melon surfaces and tomatoes likely contribute to these differing outcomes in reductions in surviving pathogen loads for GNP and UG versus CON [23,24]. Similarly, research evaluating the application of a sanitizer prior to pathogen contamination (i.e., Scenario 3) has not been extensively reported in the literature, despite the potential for post-harvest packing facilities to suffer loss of environmental control of sanitary conditions following crop washing/rinsing [25].

Sanitizing treatments modestly reduced tomato-contaminating groups of microbial hygiene indicator bacteria, producing reductions of 1.3–2.4 log_10_-cycles in a treatment-dependent manner. Like the results for pathogen-inoculated tomatoes, GNP treatment was the most effective of treatments for reducing the numbers of surviving aerobic bacteria, LAB, and coliforms (Table 3). Mesophilic aerobic bacteria and *Enterobacteriaceae* organisms both increased by <1.0 log_10_ CFU/g over 6 days of storage at 25 °C, comparable to the reductions gained here against aerobic bacteria and coliforms by GNP treatment. Smid et al. [26] reported similar reductions in epiphytic bacterial and fungal microbes on tomato surfaces following application of 13 mM *trans*-cinnamaldehyde by washing for 10 min at ambient temperature, a higher degree of reduction than was achieved by UG treatment herein. This likely occurred due to the much longer contact washing period (10 min) for the previous study than was used here (2 min), providing for longer contact of antimicrobial with microbes on tomato surfaces. Reductions achieved by HOCl treatment here were also like those reported elsewhere by others evaluating tomato sanitization by use of chlorine [19,27]. Buendía-Moreno et al. [28] recently deployed essential oil-loaded cyclodextrin capsules in a coating applied to cardboard boxes for decontaminating tomatoes during post-harvest packing. Changes in visual appearance of tomatoes were impacted by sanitization treatment, with color lightening for all treated samples, albeit to differing degrees. GNP treatment produced the greatest degree in color change, although visual appearance was not assessed for consumer acceptability or checked by objective color assessment. Nonetheless, these results indicate that while GNPs provide for useful reduction of pathogenic and hygiene-related microorganisms, they do not protect effectively against significant visual quality loss, a surprising result when compared to previous data reported by our group using the same GNP treatment against fresh spinach leaves [11].

Finally, the interaction of sanitizing treatment by experimental scenarios was statistically significant, similar to recently reported data by our group on melons [9]. As was the case there, nanoparticle encapsulation of geraniol resulted in greater reductions in numbers of surviving pathogens on treated tomato skins. This is likely the result of encapsulation enhancing the delivery of the PDA geraniol to the bacterial pathogens on tomato skin surfaces [12], regardless of the sequence of pathogen contamination and sanitization treatment. While Scenarios 2 and 3 are unlikely to be encountered in facilities adhering strictly to GAPs and rigorous sanitation programs, data reported here demonstrated improved protection (versus other treatments) against pathogen growth on treated tomatoes even when contamination occurs following sanitizing treatment.

## 5. Conclusions

Tomatoes have contributed to the onset of human foodborne disease multiple times in the U.S. and in other countries due to their ability to transmit contaminating pathogens to consumers. Sanitization of tomatoes by innovative sanitizing technologies, such as PDA-loaded nanoparticles, can effectively decontaminate tomatoes from enteric pathogens by reducing their numbers to potentially non-infectious counts. The use of encapsulation in polymeric micelles such as those reported here also represents a more effective option for reducing pathogen loads on fruit surfaces versus other tomato sanitization interventions, even in situations where cross-contamination occurs after sanitizing treatment, due to the extended slow release of the antimicrobial payload from encapsulates [12]. Such antimicrobial interventions, despite not being commonly used in the fresh produce industry, indicate an opportunity for novel produce surface sanitizers to supply the fruit and vegetable industries with useful tools to continue to protect food safety in the future.

## Figures and Tables

**Table 1:** *Escherichia coli* O157:H7 survivors (log_10_ CFU/cm^2^) on tomato skin samples by the sanitization treatment × experimental contamination/sanitization scenario interaction.

Sanitizing Treatment ^1^	Scenario 1 ^2^	Scenario 2	Scenario 3
GNP	1.82 F ^3^	3.29 DE	2.77 E
UG	4.10 ABCD	4.55 AB	3.61 CDE
HOCl	4.01 BCD	4.49 AB	3.55 CDE
CON	4.15 ABC	4.91 A	3.61 CDE
*p* = 0.0006; Pooled SE = 0.18			

^1^ GNP: 0.5 wt.% geraniol in 0.5% Pluronic F-127 nanoparticle; UG: 0.5 wt.% unencapsulated geraniol; HOCl: 200 mg/L hypochlorous acid, pH 7.0; CON: sterile distilled water wash. Treatments were applied for 2 min by immersion followed by draining of treatment fluids and sample placement in sterile covered dishes. ^2^ Treatment scenarios were designed to inoculate the organism prior to sanitizing treatment (1), inoculate prior to treatment, and again after 3 days’ storage at 5 °C (2), or inoculate after sanitizing treatment and 3 days’ storage at 5 °C (3). ^3^ Values depict means from three independent replicates (*n* = 3); means not sharing letters (A, B, C, …) differ by 2-way analysis of variance and Tukey’s Honestly Significant Differences (HSD) test at *p* = 0.05.

**Table 2:** *Salmonella typhimurium* survivors (log_10_ CFU/cm^2^) on tomato skin samples by the sanitization treatment × experimental contamination/sanitization scenario interaction.

Sanitization Treatment ^1^	Scenario 1 ^2^	Scenario 2	Scenario 3
GNP	1.86 F ^3^	3.24 DE	2.69 EF
UG	4.47 BC	4.55 B	3.51 DE
HOCl	4.52 BC	4.58 B	3.55 DE
CON	5.09 AB	5.57 A	3.61 CD
*p* =< 0.0001; Pooled SE = 0.19			

^1^ GNP: 0.5 wt.% geraniol in 0.5% Pluronic F-127 nanoparticle; UG: 0.5 wt.% unencapsulated geraniol; HOCl: 200 mg/L hypochlorous acid, pH 7.0; CON: sterile distilled water wash. Treatments were applied for 2 min by immersion followed by draining of treatment fluids and sample placement in sterile covered dishes. ^2^ Treatment scenarios were designed to inoculate the organism prior to sanitizing treatment (1), inoculate prior to treatment, and again after 3 days’ storage at 5 °C (2), or inoculate after sanitizing treatment and 3 days’ storage at 5 °C (3). ^3^ Values depict means from three independent replicates (*n* = 3); means not sharing letters (A, B, C, …) differ by 2-way analysis of variance and Tukey’s Honestly Significant Differences (HSD) test at *p* = 0.05.

**Table 3:** Least squares means of surviving aerobic bacteria, lactic acid bacteria, and coliforms following sanitization treatment application on non-inoculated tomato skin samples.

Sanitization Treatment ^1^	APC ^2^	LAB	Coliforms
GNP	3.61 A ^3^	3.39 A	1.13 A
UG	4.44 A	4.28 B	1.47 A
HOCl	5.04 AB	4.82 B	2.23 AB
CON	5.99 B	5.77 B	3.29 B
	*p* =< 0.00001; SE = 0.45	*p* = 0.0004; SE = 0.43	*p* = 0.0054; SE = 0.55

^1^ GNP: 0.5 wt.% geraniol in 0.5% Pluronic F-127 nanoparticle; UG: 0.5 wt.% unencapsulated geraniol; HOCl: 200 mg/L hypochlorous acid, pH 7.0; CON: sterile distilled water wash. Treatments were applied for 2 min by immersion followed by draining of treatment fluid and sample placement in sterile covered dishes. ^2^ APC: aerobic bacteria enumerated on 3M™ Petrifilm™ Aerobic Count Plates after 48 h incubation at 36 ± 1 °C; LAB: lactic acid bacteria on 3M Petrifilm Aerobic Count Plates inoculated with cells diluted in de Man, Rogosa, and Sharpe (MRS) broth and incubated 48 h incubation at 36 ± 1 °C; Coliforms: total coliforms enumerated on 3M Petrifilm *E. coli*/Coliform count plates after 48 h incubation at 36 ± 1 °C. ^3^ Values depict means from three independent replicates (*n* = 3); means not sharing letters (A, B) differ by one-way analysis of variance and Tukey’s Honestly Significant Differences (HSD) test at *p* = 0.05.

## Data Availability

Microbiological data presented in this manuscript are on deposit with the Texas Data Repository at https://doi.org/10.18738/T8/NXSFKB (accessed on 23 December 2021).
